# Differences in proliferation rate between CADASIL and control vascular smooth muscle cells are related to increased TGFβ expression

**DOI:** 10.1111/jcmm.13534

**Published:** 2018-03-13

**Authors:** Mahmod Panahi, Naeimeh Yousefi Mesri, Eva‐Britt Samuelsson, Kirsten G. Coupland, Charlotte Forsell, Caroline Graff, Saara Tikka, Bengt Winblad, Matti Viitanen, Helena Karlström, Erik Sundström, Homira Behbahani

**Affiliations:** ^1^ Karolinska Institute Department of Neurobiology, Care Sciences and Society Division of Neurogeriatrics Center for Alzheimer Research Huddinge Sweden; ^2^ Division of Neurodegeneration Huddinge Sweden; ^3^ Department of Geriatric Medicine Genetics Unit Karolinska University Hospital Stockholm Sweden; ^4^ Medicum Biochemistry/Developmental Biology Meilahti Clinical Proteomics Core Facility University of Helsinki Helsinki Finland; ^5^ Folkhälsan Institute of Genetics Helsinki Finland; ^6^ Department of Geriatrics Turun Kaupunginsairaala University Hospital of Turku University of Turku Turku Finland; ^7^ Karolinska Institute Department of Neurobiology, Care Sciences and Society Division of Clinical Geriatrics Karolinska University Hospital Huddinge Sweden; ^8^ Stockholms Sjukhem R&D unit Stockholm Sweden

**Keywords:** CADASIL, endothelial cells, NOTCH3, Transforming growth factor‐β, vascular smooth muscle cells

## Abstract

Cerebral autosomal‐dominant arteriopathy with subcortical infarcts and leukoencephalopathy (CADASIL) is a familial fatal progressive degenerative disorder. One of the pathological hallmarks of CADASIL is a dramatic reduction of vascular smooth muscle cells (VSMCs) in cerebral arteries. Using VSMCs from the vasculature of the human umbilical cord, placenta and cerebrum of CADASIL patients, we found that CADASIL VSMCs had a lower proliferation rate compared to control VSMCs. Exposure of control VSMCs and endothelial cells (ECs) to media derived from CADASIL VSMCs lowered the proliferation rate of all cells examined. By quantitative RT‐PCR analysis, we observed increased Transforming growth factor‐β (TGFβ) gene expression in CADASIL VSMCs. Adding TGFβ‐neutralizing antibody restored the proliferation rate of CADASIL VSMCs. We assessed proliferation differences in the presence or absence of TGFβ‐neutralizing antibody in ECs co‐cultured with VSMCs. ECs co‐cultured with CADASIL VSMCs exhibited a lower proliferation rate than those co‐cultured with control VSMCs, and neutralization of TGFβ normalized the proliferation rate of ECs co‐cultured with CADASIL VSMCs. We suggest that increased TGFβ expression in CADASIL VSMCs is involved in the reduced VSMC proliferation in CADASIL and may play a role in situ in altered proliferation of neighbouring cells in the vasculature.

## 1 INTRODUCTION

Cerebral autosomal‐dominant arteriopathy with subcortical infarcts and leukoencephalopathy (CADASIL) is the most common form of hereditary small vessel disease and is the most common genetic cause of stroke and vascular dementia in adults.^1^, ^2^ The clinical presentation of CADASIL is characterized by migraine with aura, transient neurological symptoms, mood disturbances and cognitive impairment.^3^, ^4^, ^5^, ^6^, ^7^ Pathological characteristics evident in the brain of patients with CADASIL include deposits of granular osmiophilic material (GOM) in close vicinity to the basement membrane that surrounds vascular smooth muscle cells (VSMCs) of parenchymal arterioles,^8^, ^9^ and degeneration of VSMCs 10, 11 in small and medium‐sized arteries.^12^, ^13^ Subsequent to VSMC degeneration, fibrotic thickening of the vessel walls and narrowing of the lumina of small penetrating arterioles occur predominantly in cerebral white matter.^14^ The resulting blood flow restriction leads to characteristic ischaemic changes, white matter loss and lacunar infarcts.^14^ White matter changes observed via magnetic resonance imaging (MRI) are a characteristic feature of CADASIL that have been utilized as an indication of the disease.^15^, ^16^

CADASIL is caused by mutations in the *NOTCH3* gene. NOTCH3 is a type I transmembrane receptor belonging to the Notch signalling family, one of a group of “elite” intracellular signalling pathways.^17^, ^18^ Currently, more than 230 different CADASIL‐causing mutations in the *NOTCH3* gene have been detected,^19^ which are either missense mutations or small in‐frame deletions in the epidermal growth factor repeats of *NOTCH3*. These mutations affect the number of cysteine residues, leading to an unpaired cysteine residue.^20^, ^21^, ^23^, ^24^

In CADASIL, pathological observations have shown abnormality in numerous cells within neurovascular units, including VSMCs and endothelial cells (ECs).^25^, ^26^ Therefore, alterations to each cell type within the neurovascular unit can affect the vulnerability of blood vessels in CADASIL.^25^ In CADASIL, the outer layer of the vessels consisting of VSMCs becomes thinner, while the endothelial layer that is surrounded by the VSMCs becomes swollen and loses its integrity due to gap junction loss.^25^, ^26^ This leads to microbleeds, occlusion and/or thrombosis.^27^, ^28^

It has been proposed that the degeneration of the smooth muscle layer in arteries/arterioles of patients with CADASIL is the result of increased VSMC death.^11^, ^29^ There is meagre evidence that the thinning of the tunica media is due to VSMC loss or degeneration by cell death. In a recent study, we suggested that the reduced number of cells is rather due to impaired VSMC proliferation.^29^ We showed that VSMCs from CADASIL patients with the R133C mutation in *NOTCH3* have a lower proliferation rate than control VSMCs.^29^

Transforming growth factor‐β (TGFβ) is a cytokine that has multicellular function in the cells and has a great involvement in the fibrosis of several diseases. The role of TGFβ in cell proliferation is well‐established.^30^ Dysregulation of the TGFβ signalling pathway has been proposed in CADASIL by showing increased levels of the TGFβ pro‐domain.^31^ Furthermore, latent TGFβ binding protein 1 (LTBP‐1) has been found co‐localized with NOTCH3 extracellular domain deposits in CADASIL tissues. Transforming growth factor‐β signalling has also been demonstrated to play a role in the promotion and maintenance of the contractile phenotype of VSMCs. Three TGFβ isoforms (TGFβ1, TGFβ2 and TGFβ3) have been identified in mammals, which are encoded by different genes but have high structural homology. Transforming growth factor‐β binds to membrane receptors bearing serine/threonine kinase activity, namely TGFβ receptors (TGFβR). The TGFβ isoforms have similar cellular signalling targets on the TGFβ receptors which exist in I, II, III and V subtypes.^32^ The expression level of TGFβ‐receptors is important for the TGFβ signalling pathway.^33^

This study aimed to investigate why VSMC proliferation is impaired in CADASIL. Using immortalized human umbilical, placental and cerebral VSMCs derived from individuals carrying the R133C mutation or control *NOTCH3*, we report a role of TGFβ in VSMC and EC proliferation rates, suggesting it is a critical mechanism involved in CADASIL pathophysiology.

## 2 MATERIAL AND METHODS

### 2.1 Cell lines and genotyping

Patient‐derived VSMC cell lines: umbilical cord (Umb)‐ and placental (Pla) VSMCs were established from blood vessels of genetically verified patients with CADASIL and control subjects as previously described.^34^, ^35^ The genotyping of the cerebral arterial (Cer) VSMC cell line 35 was also verified (Figure S1A).

### 2.2 Cell culture

Patient‐derived VSMC cell lines: Umb‐,^29^ Pla‐ and CerVSMC and human foreskin fibroblasts were cultured as described previously.^35^, ^36^ Umbilical artery smooth muscle cells (UASMC, Lonza) were cultured in smooth muscle cell medium BulletKit according to the manufacturer's instructions or cultured in M 231 medium with smooth muscle growth supplement (Life Technologies). Human aortic ECs (Life Technologies) were cultured in M 200 medium with low serum growth supplement (ThermoFisher Scientific).

### 2.3 Cell proliferation rate examination

CellTrace carboxyfluorescein succinimidyl ester (CFSE) (Invitrogen) was used to enable the measurement of VSMC proliferation. Carboxyfluorescein succinimidyl ester is a membrane permeant fluorescein‐based dye that can be used to track cell division due to the progressive halving of the fluorescence intensity of the dye in cells after each division. Briefly, CADASIL and control VSMC were grown to 90% confluence and harvested after washing twice with PBS. The cells were centrifuged and re‐suspended with 1 mL pre‐warmed PBS followed by labelling with 1 μmol/L CFSE for 10 minutes at 37°C. Equal numbers of CFSE‐labelled cells were incubated for either 3 or 7 days. Unlabelled cells were used as negative controls. Flow cytometry was performed using a FACSCalibur^™^ Cytometry, and data were analysed using the CellQuest software (BD Biosciences). The proliferation rate of VSMCs was also investigated using immunofluorescent staining of the proliferation‐associated Ki67 protein as previously described.^29^ The quantification of Ki67 positive cells was based on the evaluation of at least 500 cells.

### 2.4 Exposure of fibroblasts and VSMC to CADASIL PlaVSMC‐conditioned medium

Human fibroblasts and UASMC were exposed to media that CADASIL PlaVSMC cells were grown in conditioned medium for 24 hour, and 3 and 7 days. The PlaVSMC‐conditioned medium was applied either undiluted or diluted 1:1 with complete medium. The proliferation rate of VSMCs was investigated using immunofluorescence staining of the proliferation‐associated Ki67 protein as described previously.^29^ The ratio of Ki67 positive cells was based on the evaluation of at least 500 cells. Experiments were performed in triplicate.

### 2.5 Quantitative real‐time polymerase chain reaction (qRT‐PCR) of genes related to cell proliferation

RNA was isolated from Pla‐ and CerVSMC using the RNeasy Mini kit (QIAGEN). The quality of RNA was determined with RIN (RNA Integrity Number) of 10 (Figure S1B). cDNA was synthesized using the SuperScript VILO cDNA Synthesis kit (ThermoFisher Scientific) according to the manufacturer's protocol. qRT‐PCR was performed using the TaqMan Array targeting human cyclins and cell cycle regulation‐associated genes, genes encoding members of the TGFβ superfamily of ligands and four endogenous control genes (ThermoFisher Scientific). The TaqMan Array Human TGFβ pathway plate (ThermoFisher Scientific) was used for co‐culture studies. All TaqMan Probe qRT‐PCR reactions were performed in triplicate in 1 × TaqMan Fast Advanced Master Mix (Life Technologies) with 30 ng of cDNA. Quantitative RT‐PCR was performed on a 7500 Fast Real‐Time PCR System (Life Technologies). The expression of genes was normalized to the endogenous control gene; HPRT1, and the RQ (Relative Quantitation) was calculated using control VSMC as a reference. Each experiment and the control value were normalized to 1.

### 2.6 Transforming growth factor‐beta (TGFβ) antibody

Transforming growth factor‐β pan‐specific neutralizing antibody was derived from recombinant human TGFβ1, porcine platelet‐derived TGFβ1.2, porcine platelet‐derived TGFβ2 and recombinant amphibian TGFβ5 porcine platelet‐derived TGFβ1 and 2, which detects TGFβ1,TGFβ1.2, TGFβ2, TGFβ3 and TGFβ5 (AB‐100‐NA, R&D systems). Vascular smooth muscle cells was seeded at 5 × 10^4^ cells/mL in 24 well plates and cultured overnight. The medium was then changed and cells were incubated in the presence or absence of 12 μg/mL TGFβ‐neutralizing antibody for 24 hour. Vascular smooth muscle cells or ECs were fixed and stained with Ki67 antibody as described earlier.

### 2.7 Co‐culture of VSMCs and ECs in transwell system

To examine the effect of TGFβ produced by VSMC on EC proliferation rates, Pla‐ and CerVSMC were co‐cultured with ECs in a non‐contacting co‐culture transwell system (Pore size 0.4 μm, Life Technologies). Endothelial cells were plated at 5 × 10^4^ cells/mL in 6‐ or 24‐well plates. Pla‐ and CerVSMCs were seeded at 5 × 10^4^ cells/mL onto the membrane of transwell cell culture inserts and allowed to grow overnight. After 24 hour, the transwell insert membrane containing the VSMCs was placed into plates containing ECs. Vascular smooth muscle cells were treated with a TGFβ‐neutralizing antibody (12 μg/mL).

### 2.8 Statistical analysis

Statistical comparison of values obtained for VSMC cell lines was analysed by one‐way ANOVA followed by Bonferroni's post hoc test. Student *t*‐test was used for two‐group comparisons. *P*‐values <.05 were considered significant. The results are representative of three independent biological replicates expressed as mean ± SEM.

## 3 RESULTS

### 3.1 Proliferation rate in VSMCs with CADASIL mutation

**Figure 1:**
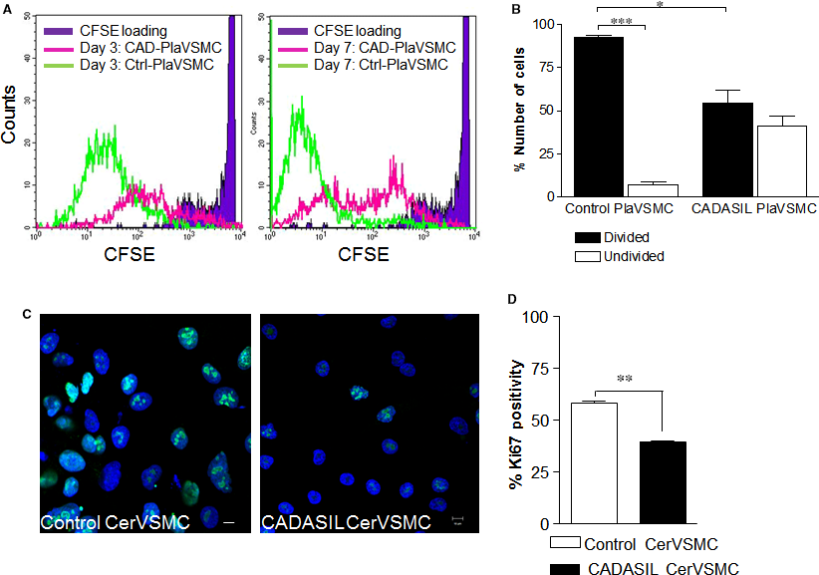
Quantification of proliferation rates in Pla‐ and CerVSMCs. A, A representative flow cytometric histogram illustrating carboxyfluorescein succinimidyl ester (CFSE) fluorescence in PlaVSMCs over 3 and 7 days. Control PlaVSMCs (green line) shows markedly decreased CFSE intensity from baseline at days 3 and 7. Cerebral autosomal‐dominant arteriopathy with subcortical infarcts and leukoencephalopathy (CADASIL) PlaVSMCs (pink line) CFSE intensity decreased over a longer period of time than in the control PlaVSMCs. B, The graph shows the proportion of divided and undivided PlaVSMCs after 3 days in culture. The undivided population represents cells with low or non‐proliferative capability (unchanged and/or higher CFSE‐staining). C, D, Representative confocal images of Ki67 staining (green) in CADASIL vs control CerVSMCs are shown. DAPI (cell nucleus; blue). Scale bar = 10 μm. ***P < .001, **P < .01,*P < .05. The results are representative of three independent biological replicates (n = 3). Student *t*‐test was used for two‐group comparisons

To examine the proliferation rate in PlaVSMC and CerVSMC, we labelled cells with CFSE dye for flow cytometry or stained with Ki67 antibody for confocal microscopy (Figure 1A‐D). A representative flow cytometric histogram illustrating CFSE fluorescence in CADASIL and control PlaVSMCs is shown in Figure 1A. Fluorescence histogram demonstrated the number of control PlaVSMCs with intense CFSE signal markedly decreased from base line (day 0) (Figure 1A, *right and left panel*), confirming that the control PlaVSMCs were actively proliferating as compared to CADASIL PlaVSMCs after 3 and 7 days (Figure 1A). Analysis demonstrated the number of CADASIL PlaVSMCs with strong CFSE fluorescence intensity indicating undivided cells was notably higher compared to control PlaVSMCs after 3 days (Figure 1B). The decrease in CFSE‐stain intensity indicating divided cells, in control PlaVSMCs compared to CADASIL PlaVSMCs, 3 days post‐CFSE labelling shows a significantly higher proliferation activity in control PlaVSMCs compared to CADASIL PlaVSMCs (Figure 1B, *P* < .05). Reduced CADASIL UmbVSMCs proliferation rate (divided) compared to control UmbVSMCs was also confirmed 29 (Figure S2, *P* < .01).

Proliferation was further evaluated by anti‐Ki67 staining and confocal microscopy (Figure 1C). A lower number of Ki67 positive cells were present for CADASIL CerVSMCs compared to control CerVSMCs (Figure 1C and D, *P* < .01).

### 3.2 The effect of CADASIL VSMC‐conditioned medium on proliferation of fibroblasts and VSMCs

**Figure 2:**
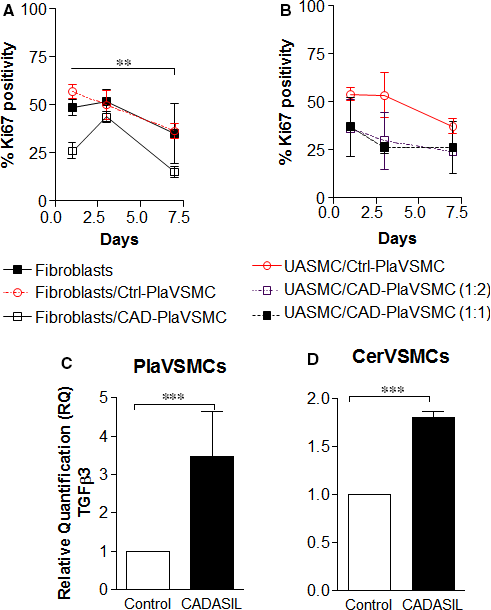
Proliferation rate of foreskin fibroblasts cultured in Cerebral autosomal‐dominant arteriopathy with subcortical infarcts and leukoencephalopathy (CADASIL) PlaVSMC‐conditioned medium. A, Human fibroblasts were incubated with PlaVSMC‐conditioned medium for 1, 3 and 7 days, stained with Ki67 antibody and analysed by confocal microscopy. B, Umbilical artery smooth muscle cells (UASMCs) were also incubated with CADASIL and control PlaVSMCs_‐_conditioned medium. C, D, qRT‐PCR analysis of *TGF*β*3* gene in Pla‐ and CerVSMCs. The expression of *TGF*β*3* gene was normalized to the endogenous control gene; HPRT1, and the RQ (Relative Quantitation) was calculated using control VSMC normalized to 1. **P < .01, ***P < .001. The results are representative of three independent biological replicates (n = 3). One‐way ANOVA followed by Bonferroni's post hoc test was used for statistical analysis

Next, we questioned whether the decreased proliferation rate observed in CADASIL VSMCs is solely an intracellular event or whether it influences neighbouring cells. Confocal microscopy and Ki67 staining demonstrated human foreskin fibroblasts cultured in CADASIL PlaVSMC‐conditioned medium displayed a lower proliferation rate when compared to fibroblasts cultured in control PlaVSMCs‐conditioned medium (Figure 2A, *P* < .01). A decreased proliferation rate was also observed when two different conditioned medium dilutions (1:1 and 1:2) from CADASIL PlaVSMCs were added to the commercial UASMC cell line (Figure 2B); however, the decrease was not significant. To assess the effect of CADASIL VSMCs on control VSMCs proliferation, we directly co‐cultured CADASIL VSMCs with control VSMCs. Overall, we observed an inhibitory effect of CADASIL VSMCs on proliferation rate of control placental and umbilical VSMCs (Figure S3A, B).

### 3.3 Elevated TGFβ gene expression in CADASIL VSMC

**Figure 3:**
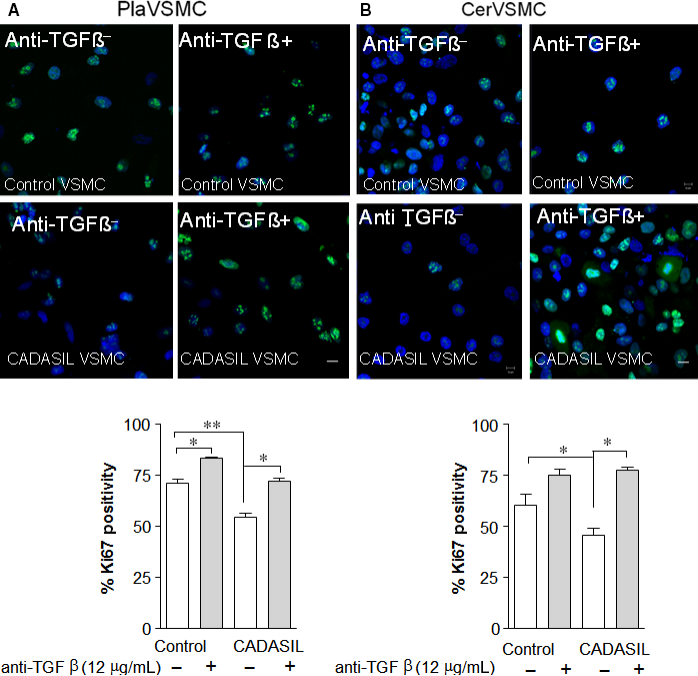
Treatment of Pla‐ and CerVSMC with Transforming growth factor‐β (TGFβ)‐neutralizing antibody. Representative confocal microscopy image of A, Pla‐ and B, CerVSMCs with and without TGFβ‐neutralizing antibody (*upper panels*), Staining; Ki67 (green), DAPI (cell nucleus; blue) and quantification analysis is shown in *lower panels*. **P < .001,*P < .05. The results are representative of three independent biological replicates (n = 3). Student *t*‐test was used for two‐group comparisons

To identify candidate factors secreted by CADASIL VSMC that could result in a lower proliferation rate of neighbouring cells, we monitored the expression level of several genes in the cell proliferation pathway by qRT‐PCR. We identified a number of genes with altered expression levels in CADASIL Pla‐ and CerVSMCs as compared to their control counterparts (Table S1, data not shown, respectively). We found an increase expression of all *TGF*β isoform families (TGFβ1, ‐2 and 3) in CADASIL VSMCs compared to control VSMCs as quantified by qRT‐PCR. However, the increased level of *TGF*β*3* was more obvious as compared to the TGFβ1, ‐2. Notably, the expression of *TGF*β*3* was significantly higher in both CADASIL Pla‐ and CerVSMCs compared to control VSMCs (Figure 2C and D, *P* < .001, respectively). Next, we investigated whether removing TGFβ would increase the proliferation rate of CADASIL VSMCs to the level of control VSMCs. Quantification analysis demonstrated a significantly lower proliferation rate of the CADASIL PlaVSMCs (*P* < .01), which addition of TGFβ‐neutralizing antibody restored to control levels (Figure 3A, *lower panel*,* P* < .05). Confocal microscopy analysis showed a significant decrease in Ki67‐positive cells in CADASIL CerVSMCs (*P* < .05), and the same effect of TGFβ‐neutralizing antibody was noted in CADASIL CerVSMCs (Figure 3B, *lower panel*,* P* < .05).

### 3.4 TGFβ secreted by CADASIL VSMC influences proliferation of endothelial cells

**Figure 4:**
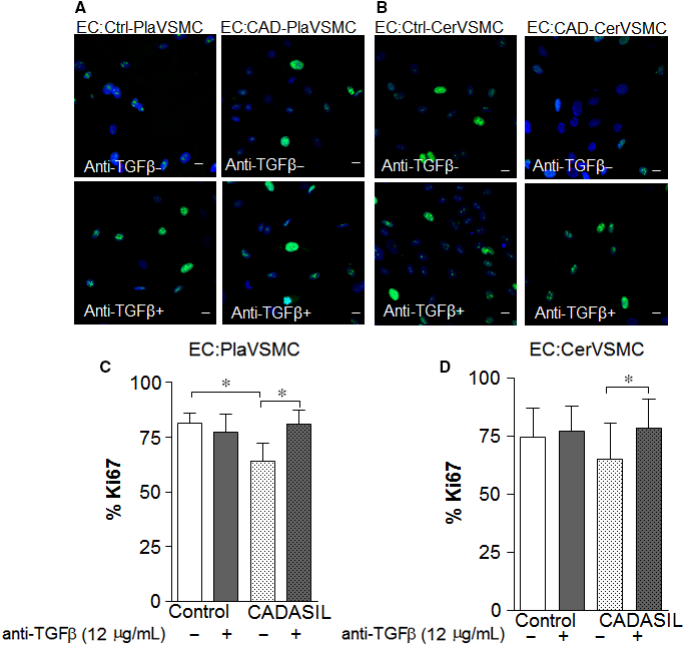
Transforming growth factor‐β secreted by Cerebral autosomal‐dominant arteriopathy with subcortical infarcts and leukoencephalopathy (CADASIL) VSMC influences proliferation of endothelial cells (ECs). A, Representative confocal images of ECs co‐cultured with PlaVSMCs and B, CerVSMCs in the presence (anti‐TGFβ+) (*lower panels*) or absence of TGFβ‐neutralizing antibody (anti‐TGFβ‐) (*upper panels*), stained with Ki67 (green). C, D, Quantitative analysis of Ki67 positive cells prior to and after TGFβ‐neutralizing antibody.*P < .05. Scale bar = 10 μm. The results are representative of three independent biological replicates (n = 3). Student *t*‐test was used for two‐group comparisons

To investigate whether TGFβ produced by CADASIL VSMC affects EC proliferation, we co‐cultured ECs with CADASIL or control Pla‐ and CerVSMCs using the transwell system (Figure 4). Endothelial cells co‐cultured with CADASIL Pla‐ and CerVSMCs exhibited a lower proliferation rate than those co‐cultured with control VSMCs (Figure 4C and D). Neutralization of TGFβ rescued the low proliferation rate of ECs co‐cultured with CADASIL Pla‐ and CerVSMCs (Figure 4A‐D, *P* < .05). The proliferation rate of ECs did not change in the presence of TGFβ‐neutralizing antibody as shown by confocal microscopy (Figure S4A) and neither did their morphology (Figure S4B).

To examine possible reasons for decreased EC proliferation, we examined the expression of *TGF*β receptors in ECs co‐cultured with CADASIL VSMCs. Our qRT‐PCR data showed that *TGF*β*R3* was down‐regulated in ECs co‐cultured with CADASIL VSMCs compared to those cultured with control VSMCs (Figure S5). *TGF*β and *TGF*β receptor 1 and 2 were unaltered by the presence of CADASIL VSMCs.

## 4 DISCUSSION

One of the major pathological findings of CADASIL is degeneration of small arterioles due to the loss of myocyte cell coverage.^11^ Previously, we have demonstrated that VSMCs derived from patient with CADASIL have a lower proliferation.^29^ In this study, we have revealed that the lower proliferation rate of these cells affects neighbouring ECs. In addition, we demonstrated that this inhibition of proliferation occurs in a paracrine fashion by inhibiting cell to cell contact using a transwell system. This paracrine mechanism seems to be due to a compound that is secreted by VSMC, and one cytokine that could potentially explain this phenomenon in CADASIL is TGFβ. By blocking this cytokine using an anti‐TGFβ antibody, we were able to restore CADASIL VSMCs and ECs co‐cultured with CADASIL VSMCs to a proliferation rate that resembles the proliferation rate of ECs co‐cultured with control VSMCs.

Transforming growth factor‐β has been shown to be involved in differentiation/proliferation of VSMCs.^30^, ^37^ Dysregulation of the TGFβ signalling pathway has been proposed to play a role in CADASIL as observed by an increased levels of the TGFβ.^31^ The TGFβ family implication in fibrosis is most interesting as fibrotic thickening of small vessels in CADASIL is widely reported.^38^ It has been suggested that the damage of VSMCs in CADASIL induces secondary fibrosis with consequent thickening of the walls and narrowing of the lumen of cerebral arteries.^19^ In this study, we found elevated expression level of *TGF*β isoforms (*TGF*β*1, TGF*β*2* and *TGF*β*3*) in Pla‐ and CerVSMCs, where the increased level of *TGF*β*3* was more obvious as compared to the *TGF*β*1* or *TGF*β*2*. TGFβ isoforms are expressed in the central nervous system and among them, TGFβ1 has been widely considered as an injury‐related cytokine which is a crucial regulator of nervous system physiology and its vasculature. TGFβ2 and TGFβ3 are also localized in radial glial cells, neuronal cell bodies in the telencephalic cortex and cerebellum, suggesting a role in the regulation of neuronal migration and differentiation as well as glial cell proliferation and differentiation. The expression of TGFβ2 and TGFβ3 persists in the entire adult CNS areas including cortex, hippocampus, striatum, brainstem and cerebellum. In the CNS, TGFβ2 mRNA and protein are predominantly expressed in astrocytes of white matter.^39^ Conclusively, the role and importance of TGFβ2 in CADASIL remain unclear. Several studies have demonstrated the ability of myofibroblasts and smooth muscle cells to express TGFβ3.^40^, ^41^, ^42^ TGFβ3 has also been detected in uninjured epithelium. However, distinct effects of vascular‐derived TGFβ3 have not been elucidated in small vessel diseases and CADASIL. In the current study, we found an up‐regulation of *TGF*β*3* gene expression in CADASIL VSMCs. Other TGFβ‐isoforms genes were also expressed higher in CADASIL VSMCs compared to control VSMCs. Variability in expression of the *TGF*β*3* gene could result in enhanced myofibroblast activity, proliferation and induction of ECM synthesis.^43^, ^44^ A previous study has identified elevated genes related to ECM proteins and collagens in CADASIL.^45^ There is a possibility that the observed increased secretion of TGFβ‐isoforms, in particular TGFβ3 in our study, could be involved in induction of ECM protein synthesis and fibrosis formation that disrupts the normal architecture of vasculature in CADASIL. Whether fibrosis is a general response to degeneration of VSMCs or because of the activation of TGFβ‐specific fibrotic pathways is still to be examined.

Degenerative changes in capillary vessels are also involving ECs in CADASIL and showing endothelial abnormalities which suggests a secondary character of endothelium damage in CADASIL.^46^ EC function in blood vessels is regulated by coordination of vascular VEGF, Notch and TGFβ.^47^ Studies have shown that signalling of TGFβ/ALK1–Smad1/5 stimulates EC migration and proliferation.^48^ Inhibition of endoglin in ECs, which is a part of the TGFβ receptor complex, potentiates TGFβ/ALK1 signalling,^49^, ^50^ resulting in reduced proliferation.^51^, ^52^ TGFβ binds to its specific receptors including TGFβR3. TGFβR3 is one of the proteins that have a more indirect role in TGFβ signal transduction.^33^ The down‐regulation of TGFβR3 on EC potentiates the inhibitory effect of TGFβ on EC migration and growth.^50^ We investigated whether *TGF*β receptor expression may have an impact on EC proliferation in ECs co‐cultured with CADASIL VSMCs. Our qRT‐PCR data showed down‐regulation of *TGF*β*R3* in ECs co‐cultured with CADASIL VSMCs (Table S2, Figure S5), while *TGF*β*R1* and *TGF*β*R2* in ECs were unaffected (data not shown). Interestingly, the presence of TGFβ‐neutralizing antibody significantly changed the *TGF*β*R3* gene expression level, which suggests that TGFβ exerts its action on EC proliferation through the *TGF*β*R3*‐receptor. This data suggest that several members of the TGFβ superfamily are potentially involved in proliferation rate changes in our system.

Several studies have demonstrated that during NOTCH target gene activation, there is an interaction between NOTCH and TGFβ signalling pathways. Both NOTCH and TGFβ are known to be involved in many regulatory functions of VSMC and other cells, such as Wnt and MAP kinase signalling, cell cycle‐related genes (*CDC34, CHEK1, etc*.).

So far, few studies have addressed gene expression and signalling pathways in CADASIL. However, it has been shown that both TGFβ and NOTCH are involved in differentiation and proliferation of VSMCs. In CADASIL, due to mutations in *NOTCH3* gene, the synergy between TGFβ and NOTCH3 might be compromised. In this study, increased TGFβ expression was observed in CADASIL *NOTCH3*‐mutated VSMCs. It could therefore be expected that also other factors involved in cell proliferation, differentiation and ECM family gene expression in VSMCs are affected. Further, changes to NOTCH3 function due to mutations and increased TGFβ in CADASIL can also have an impact on TGFβ receptor expression in ECs, as shown in our study. The interplay between TGFβ and NOTCH3 in CADASIL needs further elucidation for the understanding of the VSMCs degeneration.

We believe that increased TGFβ3 reflects an inflammatory condition and even an involvement of TGFβ in fibrosis in CADASIL. Support for our hypothesis is the outcome of several studies that showed NOTCH3 and TGFβ1 signalling play a key role in the pathogenesis and progression of chronic cardiovascular disease.^53^ NOTCH3 was shown to be an important protective factor against cardiac fibrosis in a myocardial infarction model, and the protective effect of NOTCH3 is attributable to its action on TGFβ1/Smad3 signalling. This might also occur in patients with CADASIL, where the signalling of TGFβ pathway and the effect of gene expression switch the normal function of its action. The role of activated NOTCH signalling and TGFβ has also been shown in liver fibrosis.^54^ Once the intracellular domain of the NOTCH protein (NICD) is transported into nucleus, it can interact with a number of different transcription factors, including FoxH1, c‐Jun, c‐Fos, Gli‐3 and others and control expression of a large number of target genes, including, for example, genes involved in cell cycle control, extracellular matrix regulation and mesoderm specification. The TGFβ signalling pathway is known to be involved in modulation of cyclin genes.^55^ We have also observed down‐regulation of cyclins gene expression in CADASIL VSMCs, suggesting that TGFβ signalling pathway may exert its effect on cyclin genes.

In CADASIL, the cells in the vasculature can be exposed to complex changes, such as aggregated NOTCH3, and GOM. In this study, we have implicated TGFβ and TGFβ receptors as additional players in CADASIL disease. Therefore, more detailed studies are needed to elucidate the interplay of TGFβ signalling in the vasculature in CADASIL. In summary, our data provide evidence for direct involvement of TGFβ in proliferation of VSMCs and ECs. We believe that our findings provide a better understanding of the pathogenesis of CADASIL disease.

## CONFLICT OF INTEREST

The authors have no conflicts to disclose.

## AUTHOR CONTRIBUTION

MP, LF, NM, KC, CG and EVS performed the research; MP, HK, MV, BW, ES and HB designed the research study; MP, NM, LF and HB analysed the data; MP, KC, HK, ES and HB wrote the manuscript.
