# Supplementary figures and images for: Differences in proliferation rate between CADASIL and control vascular smooth muscle cells are related to increased TGFβ expression
Source: J Cell Mol Med. 2018 Mar 13;22(6):3016–24. doi: 10.1111/jcmm.13534 (PMC5980144; doi:10.1111/jcmm.13534)

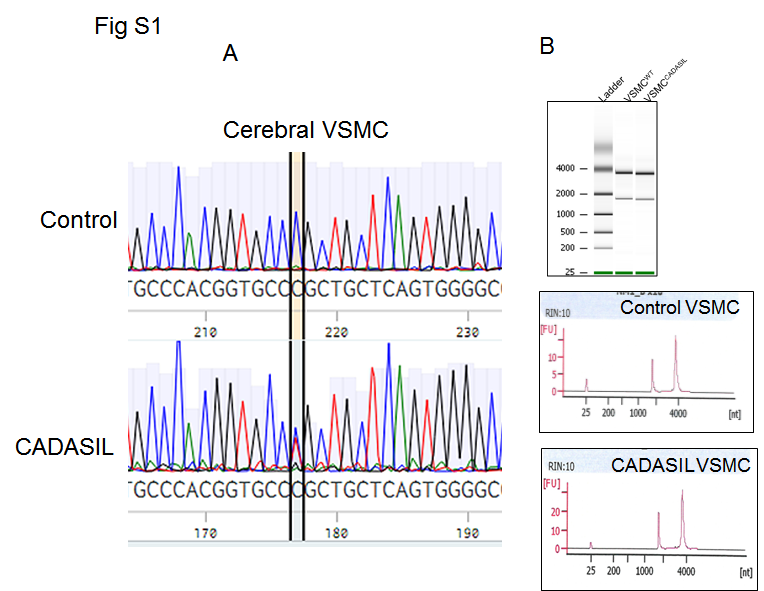

Supplement: Supplementary file 1 [file JCMM-22-3016-s001.tif]

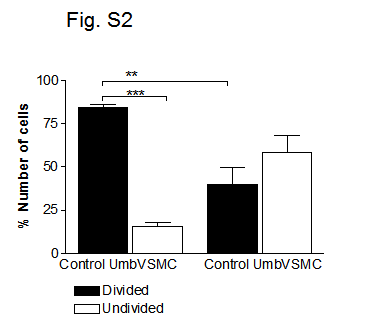

Supplement: Supplementary file 2 [file JCMM-22-3016-s002.tif]

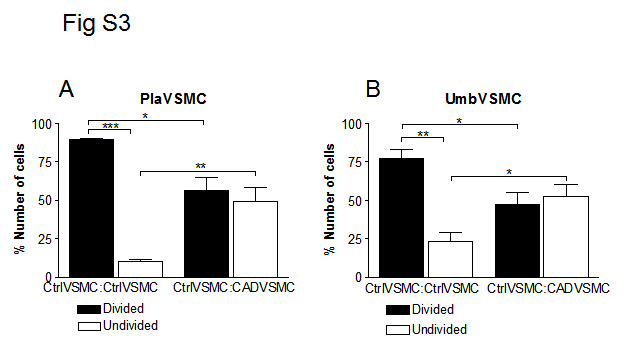

Supplement: Supplementary file 3 [file JCMM-22-3016-s003.tif]

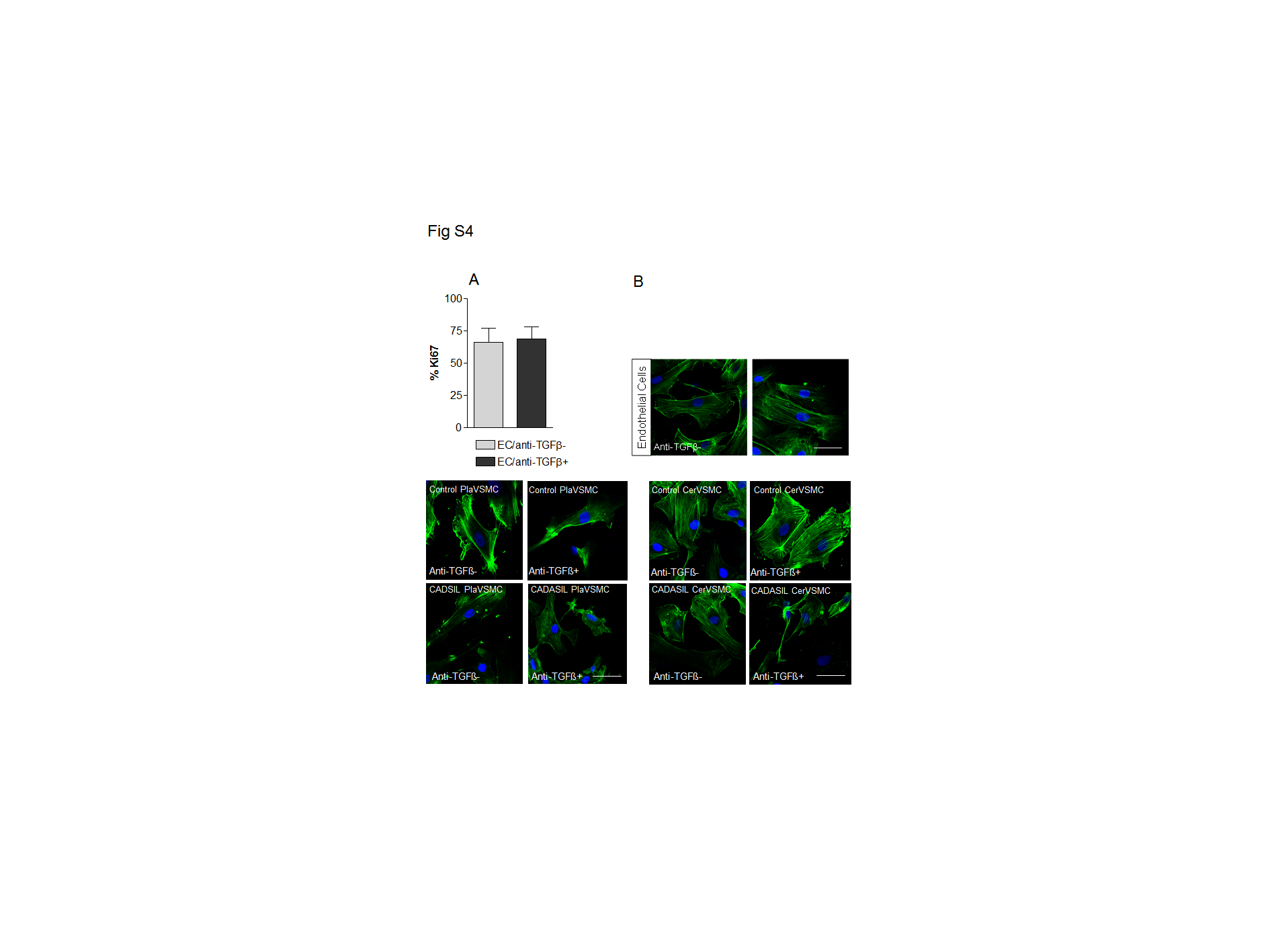

Supplement: Supplementary file 4 [file JCMM-22-3016-s004.tif]

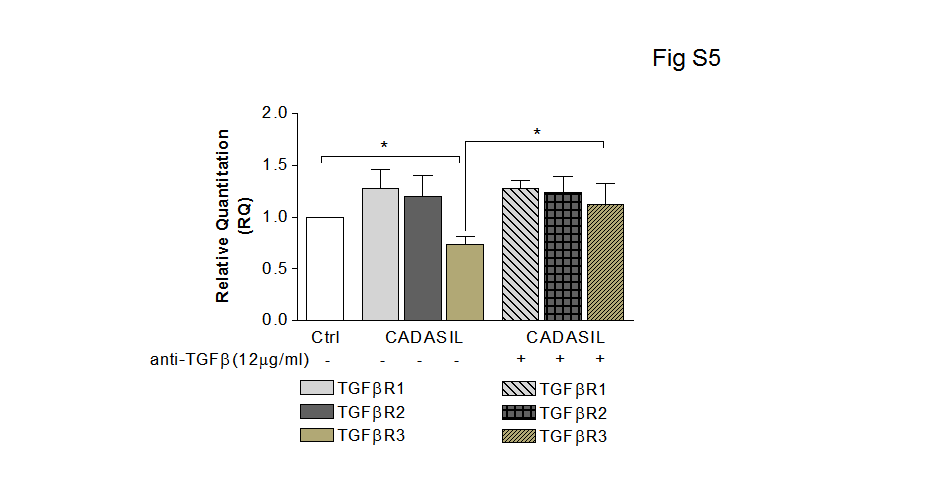

Supplement: Supplementary file 5 [file JCMM-22-3016-s005.tif]

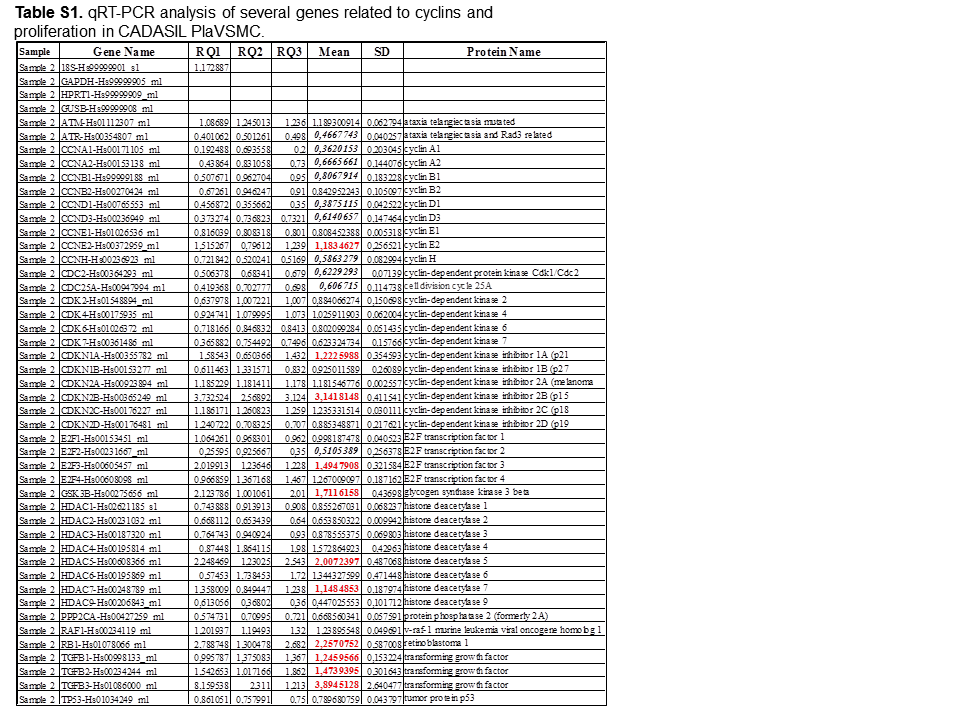

Supplement: Supplementary file 6 [file JCMM-22-3016-s006.tif]

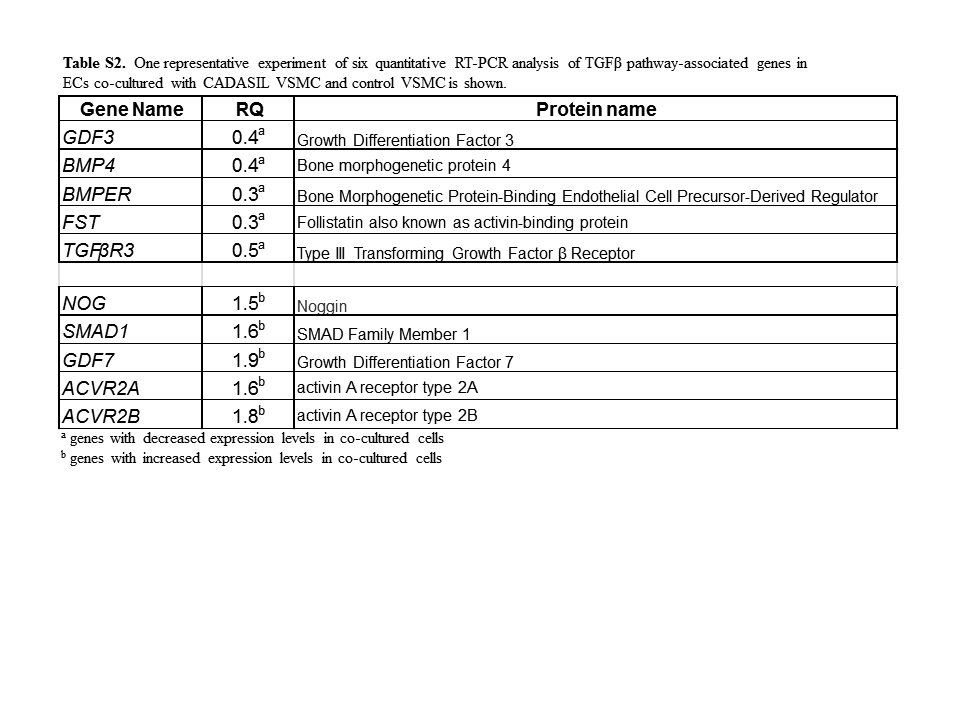

Supplement: Supplementary file 7 [file JCMM-22-3016-s007.tif]
