# Supplementary material for: Differences in proliferation rate between CADASIL and control vascular smooth muscle cells are related to increased TGFβ expression
Source: J Cell Mol Med. 2018 Mar 13;22(6):3016–24. doi: 10.1111/jcmm.13534 (PMC5980144; doi:10.1111/jcmm.13534)
Supplement: Supplementary file 8 [file JCMM-22-3016-s008.docx]

**Supporting Information**

**Appendix S1**

**S1. Materials and Methods**

**Cell genotyping.** The presence or absence of c.475C>T (p.Arg133Cys) in the cell lines was verified by amplifying exon 4 of *NOTCH3* gene by PCR and digesting the PCR product with restriction enzyme MspA1I.

**Direct co-culture of control vs CADASIL VSMCs.** For direct VSMC cell culture, the CFSE labeled CADASIL and control VSMC were seeded and cultured with an equal number of the unlabeled CADASIL or control VSMC for 3 and 7 days, harvested and prepared for flow cytometry as described above.

**Immunohistochemical staining.** For morphological studies, the ECs co-cultured with Pla- and CerVSMC in the presence or absence of anti-TGFβ, were stained with α-actin skeleton using Alexa Fluor 488 Phalloidin as described above. Confocal analysis was performed to study the effect of anti-TGFβ on the morphology of ECs co-cultured with CADASIL and control Pla- and CerVSMC. To examine EC morphology, ECs were seeded at 10 × 10^4^ cells/mL. 16 hours post-seeding cells were fixed with 4% paraformaldehyde (Sigma Aldrich) and blocked using 10% normal goat serum and 3% bovine serum albumin (BSA, Sigma Aldrich) for 40 min at 37°C. The α-actin skeleton was stained using Alexa Fluor 488 Phalloidin (Life Technologies) at a 1: 150 dilution for 30 min at 37°C. Cells were washed with PBS and mounted with Duolink mounting medium with DAPI (Sigma Aldrich). All immunofluorescent imaging was performed with a Zeiss META LM510 system.

**Supporting Information**

**S2. Figure legends**

**Figure S1 Genotyping of CerVSMCs.** The Sanger sequencing was used to verify the presence or absence of the c.47 5C ˃T (rs137852642) SNP that results in the p.Arg133Cys missense mutation. The following primer pairs covering the mutation site were used: Forward: 5’-GGGGTGTGGTCAGTCCTAAA-3’, Reverse 5’ GGAAGGAGCCAGGTGTGTT-3’.The genotyping of the cerebral VSMC cell line confirmed the c.475C >T genotype and R133C mutation in this cell line.

**Figure S2** **Proliferation rate of UmbVSMCs analyzed by flow cytometry.** Patient derived umbilical cord VSMC from CADASIL or controls were labeled with CFSE and analyzed by flow cytometry. The graph is showing the cell population of divided and undivided UmbVSMCs as determined by flow cytometric analysis of CFSE staining. The divided population presents the proliferation activity, which analysis was based on the reduced percentage of the CFSE-stained cells after 3 days in culture. The undivided population presents the cells with low or non-proliferative capability (unchanged and/or low CFSE-staining). Statistical analysis showed significant increase in the cell number of divided population of the control UmbVSMC compared to its undivided population (****P* < 0.001). After 3 days post-CFSE labeling, the divided cell population in CADASIL UmbVSMC (reduced CFSE staining), was significantly lower as compared to divided cell population of control UmbVSMC (**P* < 0.05) (*n = 3*). Values are presented as mean ± S.E.M. Student *t*-test was used for two-group comparisons.

**Figure S3 Direct co-culture of PlaVSMC with control or CADASIL VSMCs.** (**A**) CFSE labeled control PlaVSMC co-cultured with non-labeled CFSE control PlaVSMC exhibited no decrease in proliferation (****P* < 0.001), as shown by percentage number of divided cells. However, CFSE labeled control PlaVSMC cultured with CADASIL PlaVSMC displayed a significant decrease in proliferation (reduced numbers of divided cells) after 3 days (***P* < 0.05). (**B**) A similar result was observed for UmbVSMC cell line. *n* = 3, values are presented as mean ± S.E.M. Student *t*-test was used for two-group comparisons.

**Figure S4** **ECs co-culture with VSMCs.** To determine whether secreted TGFβ from VSMCs change ECs proliferation and morphology, CADASIL and control VSMCs were incubated in the presence or absence of TGFβ-neutralizing antibody. (**A**) Neutralization of TGFβ in control PlaVSMC did not alter the EC proliferation rate as analyzed by flow cytometry. (**B**) Confocal microscopy observation demonstrated that ECs morphology was not changed in response to TGFβ-neutralizing antibody. Neither, did confocal analysis reveal morphological alterations of typical EC morphology after co-culture with CADASIL or control Pla- or CerVSMC cells. No change of α-actin arrangement was observed in ECs after co-culture with CADASIL Pla- or CerVSMCs. Scale bar = 10 µm. Student *t*-test was used for two-group comparisons.

**Figure S5** **Quantitative RT-PCR analysis of *TGFβ3R* gene expression in ECs co-cultured with CerVSMCs.** To investigate which receptor was decreased (R1, R2 and R3) co-culture was performed with CADASIL CerVSMC and EC (*n = 3*). *TGFβR3* showed decreased expression **P* < 0.05, in the absence of TGFβ neutralizing antibody whereas to two other receptors were unchanged. In the presence of TGFβ neutralizing antibody the *TGFβR3* gene expression level was significantly changed, indicating a role of TGFβ on EC proliferation through the R3-receptor (**P* < 0.05). One-way ANOVA followed by Bonferroni’s post-hoc test was used for statistical analysis. Values are presented as mean ± S.E.M.

**Table S1.** **Quantitative RT-PCR analysis of genes related to cyclins and proliferation in CADASIL VSMC.** The table is showing qRT-PCR analysis of genes related to cyclins and proliferation in CADASIL PlaVSMC. The results are representative of three independent biological replicates (*n* = 3), which presenting up- or down-regulation of several genes in CADASIL VSMCs, RQ: Relative Quantitation. Values are presented as mean ± S.D.

**Table S2. Quantitative RT-PCR analysis of gene expression levels in ECs co-cultured with CerVSMCs.** A number of genes with altered expression levels in EC after co-culture with CADASIL Pla- or CerVSMC were identified. One representative experiment of six quantitative RT-PCR analyses is shown.
